# Diffusion Tensor Imaging Tractography Reveals Disrupted White Matter Structural Connectivity Network in Healthy Adults with Insomnia Symptoms

**DOI:** 10.3389/fnhum.2017.00583

**Published:** 2017-11-30

**Authors:** Feng-Mei Lu, Jing Dai, Tania A. Couto, Chun-Hong Liu, Heng Chen, Shun-Li Lu, Li-Rong Tang, Chang-Le Tie, Hua-Fu Chen, Man-Xi He, Yu-Tao Xiang, Zhen Yuan

**Affiliations:** ^1^Bioimaging Core, Faculty of Health Sciences, University of Macau, Macau, China; ^2^Chengdu Mental Health Center, Chengdu, China; ^3^The Clinical Hospital of Chengdu Brain Science Institute, MOE Key Lab for Neuroinformation, University of Electronic Science and Technology of China, Chengdu, China; ^4^Beijing Institute of Traditional Chinese Medicine, Beijing Hospital of Traditional Chinese Medicine Affiliated to Capital Medical University, Beijing, China; ^5^Department of Radiology, Beijing Anding Hospital, Capital Medical University, Beijing, China; ^6^Key Laboratory for NeuroInformation of Ministry of Education, School of Life Science and Technology, University of Electronic Science and Technology of China, Chengdu, China

**Keywords:** insomnia, diffusion tensor imaging tractography, white matter, graph theoretical analysis, small-world network

## Abstract

Neuroimaging studies have revealed that insomnia is characterized by aberrant neuronal connectivity in specific brain regions, but the topological disruptions in the white matter (WM) structural connectivity networks remain largely unknown in insomnia. The current study uses diffusion tensor imaging (DTI) tractography to construct the WM structural networks and graph theory analysis to detect alterations of the brain structural networks. The study participants comprised 30 healthy subjects with insomnia symptoms (IS) and 62 healthy subjects without IS. Both the two groups showed small-world properties regarding their WM structural connectivity networks. By contrast, increased local efficiency and decreased global efficiency were identified in the IS group, indicating an insomnia-related shift in topology away from regular networks. In addition, the IS group exhibited disrupted nodal topological characteristics in regions involving the fronto-limbic and the default-mode systems. To our knowledge, this is the first study to explore the topological organization of WM structural network connectivity in insomnia. More importantly, the dysfunctions of large-scale brain systems including the fronto-limbic pathways, salience network and default-mode network in insomnia were identified, which provides new insights into the insomnia connectome. Topology-based brain network analysis thus could be a potential biomarker for IS.

## Introduction

Insomnia is one of the most prevalent sleep disorders that is distinguished by difficulties in falling or maintaining sleep, and/or early morning awakening (Morin and Benca, 2012; Cheung et al., 2013; Morin et al., 2015; Riedner et al., 2016). Insomnia is associated with impaired daytime functioning and affects approximately one-third of the general population (Ohayon, 2002; Moore, 2012; Morin and Benca, 2012; Kronholm et al., 2015). In addition, individuals with insomnia show an increased risk for developing other psychiatric disorders. For example, nearly 40% of the insomnia patients have a comorbid psychiatric disorder, and almost all of depression patients present high risk for insomnia (Taylor et al., 2005; Kaneita et al., 2006; Ohayon and Hong, 2006; Benca and Peterson, 2008; Wulff et al., 2010; Mayer et al., 2011). More importantly, insomnia can lead to feeling fatigued, poor academic performance, working disability, drugs and alcohol abuse, suicidal thoughts and reduced quality of life (Short et al., 2013; Kronholm et al., 2015). Consequently, insomnia can negatively impact personal and public health, incur direct and indirect healthcare costs, and have a huge socio-economic impact on society (Kucharczyk et al., 2012; Moore, 2012; Lian et al., 2015). Although well-documented neuroimaging studies have investigated insomnia, the neurobiological mechanisms underlying this psychiatric disorder remain poorly understood.

Growing functional and structural neuroimaging evidence shows that widespread brain regions are implicated in the pathobiology of insomnia, including the amygdala, hippocampus, anterior cingulate gyrus, caudate nucleus, insula and the frontal areas (Drummond et al., 2004; Nofzinger et al., 2004; Riemann et al., 2007, 2015; Altena et al., 2008, 2010; Spiegelhalder et al., 2013, 2015; Winkelman et al., 2013; Baglioni et al., 2014; Joo et al., 2014; Stoffers et al., 2014; Liu C.-H. et al., 2016; Lu et al., 2017). In particular, the functional and structural networks are strongly correlated with each other, and the structural connectivity works as a physical substrate of the functional connectivity. The functional connectivity can also affect the structural connectivity according to the brain plasticity (van den Heuvel et al., 2008; Greicius et al., 2009; Rubinov et al., 2009; Zhang J. et al., 2011; Long et al., 2015). In addition, it has been widely recognized that functional interactions among different brain regions are effectively constrained by large-scale structural connections (Hagmann et al., 2008; Honey et al., 2009). However, the network-level structural deficits remain largely unknown, especially the topological alterations associated with insomnia. It is therefore essential to examine the structural substrate of interactions among distributed brain regions to understand the functional brain activation patterns in insomnia.

Diffusion tensor imaging (DTI) tractography is a robust, non-invasive method that can be utilized to reconstruct the white matter (WM) tracts of the human brain (Basser et al., 2000; Guo W.-B. et al., 2012; Guo W. et al., 2012). When combined with a graph theoretical approach, this advanced neuroimaging technique can allow us to characterize the structural connection patterns of the human brain *in vivo*. Graph theoretical analysis can delineate the whole brain as a large-scale network consisting of nodes (brain areas) and edges (functional connectivity between pairs of areas; Bullmore and Sporns, 2009). Both of these methods have been increasingly used for the reconstruction of brain WM structural connectivity networks in psychiatric disorders such as post-traumatic stress disorder (Long et al., 2013), depression (Long et al., 2015), Alzheimer’s disease (Lo et al., 2010), and multiple sclerosis (Shu et al., 2011). So far, there are only two studies exploring the integrity of WM in insomnia, one study demonstrated that insomnia is associated with reduced integrity of WM tracts in the anterior internal capsule by comparing the fractional anisotropy (FA) between 24 primary insomnia (PI) patients and 35 healthy controls (Spiegelhalder et al., 2014) and by performing the between-group comparisons of the WM tracts between 23 PI patients and 30 healthy controls, another study suggested that the insomnia patients had decreased integrity of WM tracts predominantly in the areas of right anterior and posterior limb internal capsule, right anterior and superior corona radiate and right thalamus (Li S. et al., 2016). As such, this pilot work focuses on revealing topology abnormalities in WM structural connectivity networks associated with insomnia.

In this study, we hypothesized that compared to the healthy subjects without insomnia symptoms (NIS) group, the healthy subjects with IS group would exhibit an altered structural topology and disrupted nodal network properties of the brain areas mainly involved in the fronto-limbic system, salience network, and default-mode network. The DTI data from IS group and NIS group were collected first. Then, we constructed the whole brain WM structural connectivity networks with 90 nodes represented by cerebrum brain areas using the automated anatomical labeling (AAL) template and corresponding edges defined as the mean FA using DTI tractography. In addition, we applied graph theoretical analysis to generate the small-world characteristics of these WM networks, which can be used to identify the altered topological properties of brain networks in insomnia. More importantly, we examined the associations between clinical data and the altered network topologies.

## Materials and Methods

### Participants

The study participants comprised 92 right-handed healthy subjects (female/male: 51/41, age: 20–60 years). All participants were first screened with the Non-Patient Structured Clinical Interview for the Diagnostic and Statistical Manual of Mental Disorders (DSM-IV; SCID) by two independent experienced psychiatrists (LRT and CLT) as described in our previous study (Lu et al., 2017). All the subjects had no neurological or psychiatric disorders, such as depression, anxiety disorders, epilepsy, schizophrenia, mental retardation, or chronic pain. In addition, none of the subjects had taken any psychotropic medication in at least 2 months prior to the MRI scans.

All clinical tests were approved by the Medical Ethics Committee of Beijing Anding Hospital, Capital Medical University, the Imaging Center for Brain Research of Beijing Normal University and the Biomedical Ethics Board of the Faculty of Health Sciences at the University of Macau (Macao SAR, China) in accordance with the approved guidelines. All subjects gave written informed consent in accordance with the Declaration of Helsinki. The study groups included 30 healthy subjects with IS (age: 38.00 ± 11.85 years) and 62 healthy subjects without IS (age: 37.47 ± 11.95 years). We find no significant differences in gender, age, as well as educational level between two groups. Table 1 provides the demographic characteristics of the participants.

**Table 1:** Demographic and clinical data.

Measurements	IS (*N* = 30)	NIS (*N* = 62)	*t*	*p*-value
Age (years)	38.00 ± 11.85	37.47 ± 11.95	0.201	0.841^a^
Gender (M/F)	15/15	26/36	0.532	0.466^b^
Education level (years)	14.07 ± 3.34	15.42 ± 2.95	−1.889	0.065^a^
Handedness (R/L)	30/0	62/0	-	-
HAMD score	2.93 ± 1.46	0.19 ± 0.51	9.987	<0.001^a^
Adjusted HAMD score	1.23 ± 1.31	0.19 ± 0.51	4.214	<0.001^a^
HAMA score	3.20 ± 2.12	0.32 ± 0.79	7.187	<0.001^a^
Adjusted HAMA score	1.97 ± 2.00	0.32 ± 0.79	4.327	<0.001^a^
Insomnia score	1.70 ± 0.92	0.00 ± 0.00	10.172	<0.001^a^

*Data are presented as mean ± SD. Adjusted HAMD score means HAMD scores after omission of sleep questions. Adjusted HAMA score means HAMA scores after omission of sleep questions. M, male; F, female; R, right; L, left; IS, healthy subjects with insomnia symptoms; NIS, healthy subjects without insomnia symptoms; SD, standard deviation; HAMD, Hamilton Depression Rating Scale; HAMA, Hamilton Anxiety Rating Scale. ^a^The *p* value was obtained by two sample* t*-tests. ^b^The *p* value for gender distribution in the two groups was obtained by chi-square test*.

### Insomnia Symptoms Measurements

The 17-item Hamilton Depression Rating Scale (HAMD-17) was used to measure the severity of participants’ IS (Hamilton, 1967), which is based on the sum of the three items on the sleep subscale of HAMD-17. A total score greater than or equal to one indicated IS. The severity of depression and anxiety in all the subjects was also assessed using HAMD-17 and the Hamilton Anxiety Rating Scale (HAMA). In the current study, the adjusted HAMD and adjusted HAMA scores were generated by omitting the insomnia-related items to prevent a potential influence of IS from these scales on our findings (Lu et al., 2017). Clinical data for the two groups are given in Table 1.

### Image Acquisition

All participants were scanned with a 3T Trio MRI scanner (Siemens Medical Solutions, Erlangen, Germany) located in the National Key Laboratory for Cognitive Neuroscience and Learning, Beijing Normal University. Foam padding and earplugs were used for all subjects in order to reduce the head motion. During the data recording, all participants were instructed to completely relax without thinking of particular things, rest quietly, close their eyes, remain still and keep awake. T_1_-weighted, sagittal 3D magnetization-prepared rapid gradient-echo (MP-RAGE) sequences were acquired with the following scanning parameters: repetition time (TR) = 2530 ms; echo time (TE) = 3.39 ms; matrix = 256 × 256; slices = 128; flip angle = 7°; field of view (FOV) = 256 × 256 mm^2^; slice thickness = 1.33 mm; inter-slice gap = 0 mm; voxel size = 1 × 1 × 1.33 mm^3^; orientation = sagittal. DTI data were collected using a single-shot echo-planar imaging (EPI) sequence: TR = 7200 ms; TE = 104 ms; matrix = 128 × 128; slices = 49; flip angle = 90°; FOV = 128 × 128 mm^2^; slice thickness = 2.5 mm; inter-slice gap = 0 mm; voxel size = 1.8 × 1.8 × 2.5 mm^3^; orientation = axial; 64 non-collinear diffusion weighting gradient direction (*b* = 1000 s/mm^2^) and one additional image without diffusion weighting (*b* = 0 s/mm^2^).

### Structural Network Construction

The structural networks were constructed for all participants. The network nodes were characterized by the brain areas divided by the AAL template (Tzourio-Mazoyer et al., 2002), whereas the network edges were defined as fiber tracts that linked with these nodes.

#### Definition of Network Nodes

The procedure to define network nodes was according to the previous study (Gong et al., 2009). The regions of interest (ROIs) were described in diffusion native space. In brief, each subject’s T_1_-weighted image was first co-registered to the non-diffusion-weighted (*b* = 0 s/mm^2^) images in the diffusion native space through a linear transformation. Then the co-registered T_1_ images were non-linearly converted to the ICBM-152 T_1_-template in the Montreal Neurological Institute (MNI) space. The 12 degrees of freedom combined with nonlinear warps were applied in this step. The inverse transformation parameter was used to warp the AAL areas from MNI space to the DTI native space by using a nearest-neighbor interpolation method based on the statistical parametric mapping (SPM8) package. Using this procedure, 90 cortical and subcortical brain areas were generated (45 for each hemisphere, see Supplementary Table S1).

#### WM Tractography

The following steps were carried out to reconstruct the whole-brain WM tracts. The distortions were corrected for the effects of eddy current with an affine alignment of the diffusion-weighted images to the non-diffusion-weighted images based on the FMRIB Diffusion Toolbox (FSL)^1^. Subsequently, the diffusion tensor matrix was generated on a voxel-by-voxel analysis, which was further diagonalized to generate three eigenvalues and associated eigenvectors. The diffusion tensor models were calculated using the linear least-squares fitting algorithm at the voxel level by using the Diffusion Toolkit (Wang et al., 2007). The DTI fiber tracking procedures were carried out in diffusion native space based on the Fiber Assignment by Continuous Tracking (FACT) method through the Diffusion Toolkit (Wang et al., 2007). All of the path tracing in the dataset terminated if either the FA of each voxel did not exceed 0.2 or the tracking angle was greater than 45 degrees (Shu et al., 2011).

#### Definition of Network Edges

To determine the brain network edges in the native diffusion space, an ROI *i* and ROI *j* were considered to be linked through an edge where at least one fiber was present between them (Gong et al., 2009). The connections were weighted by the mean FA values of fibers that connected these two ROIs to depict the connectivity strength between ROI *i* and ROI *j*.

### Network Analysis

#### Threshold Selection

Each correlation matrix was thresholded into a set of undirected binary networks by applying a threshold of sparsity *S*, which was computed as the ratio of the total number of existing edges with the all possible total number of edges. Then the number of nodes and edges of these normalized resulting networks were the same which made it possible to explore the between-group differences in regard to network topological organization (Bullmore and Bassett, 2011). Each connectivity matrix was thresholded over a threshold range of 0.1 ≤ *S* ≤ 0.2 with intervals 0.01 repeatedly.

The minimum threshold (*S* = 0.1) was determined according to the criterion that the averaged degree of all network nodes at each thresholded should be larger than 2log(N), in which N was 90 here denotes the total number of nodes. All individual networks reached 90% full connections at the minimum threshold. The maximum threshold (*S* = 0.2) was computed by obtaining the individual network topological cost without being thresholded, and then selected the minimum sparsity threshold (Long et al., 2013).

#### Small-World Properties

To measure the small-world properties of constructed structural brain networks, we first produced 100 random networks by using a Markov-chain algorithm and each random network has the same number in regard to nodes, edges and degree distribution with a real brain network (Liao et al., 2010). A real brain network can be regarded as a small world network only if it satisfied the conditions of both γ > 1 and λ ≈ 1 (Watts and Strogatz, 1998), or sigma *σ* = λ/γ > 1, which indicated that a small-world network possess a higher clustering coefficient and a similar path length as compared with a random network (Humphries et al., 2006; Liu et al., 2008). Typically, we scaled the characteristic shortest path length *L*_p_ and the clustering coefficient *C*_p_ of the constructed structural networks with the averaged *L*_random_ and *C*_random_ of all 100 random networks (i.e., the normalized characteristic path length, λ=LpLrandom and the normalized clustering coefficient, γ=CpCrandom), where *L*_random_ and *C*_random_ denote the averaged characteristic shortest path length and the averaged clustering coefficient of 100 generated random networks, respectively.

#### Network Metrics

To evaluate the nodal properties of cortical and subcortical brain regions in structural networks, three key measurements were computed: the nodal degree *Deg*_i_, the nodal efficiency *E*_i_, and the nodal betweenness *BC*_i_. Additionally, the global efficiency *E*_glo_ and local efficiency *E*_loc_ were used to define the network efficiency (Achard and Bullmore, 2007). We first computed the six global network properties *C*_p_, *L*_p_, *λ*, *γ*, *E*_glo_, and *E*_loc_, and three regional nodal parameters *Deg*_i_, *E*_i_ and *BC*_i_. Furthermore, we computed the area under the curve (AUC) of each parameter, which denoted an integrated index for the topological organization of brain networks (Zhang Z. et al., 2011; Liu F. et al., 2016). Detailed information about the network properties is provided in the Supplementary Materials.

### Statistical Analysis

#### Differences in the Network Properties

To show the differences of the global network topological properties between the two groups, nonparametric permutation tests (5000 iterations, *p* < 0.05, uncorrected) were carried out for each network topology over the threshold of 0.1 ≤ *S* ≤ 0.2 with intervals of 0.01 and the AUCs (Bullmore et al., 1999) of each network topology. In addition, nonparametric permutation tests (5000 iterations, *p* < 0.05, uncorrected) were also carried out on the AUCs of each regional nodal property to determine whether there were significant group distinctions between groups. Before performing the permutation tests, a multiple regression analysis was conducted to regress out the gender, age, educational level, adjusted HAMA score and adjusted HAMD score as dependent variables to exclude the influence of the depression and anxiety and the independent variable is the AUC of each network metric. A value of *p* < 0.05 was considered uncorrected significant for multiple regression analysis.

#### Correlations between the Network Properties and Insomnia Scores

We also examined the relationships between the global and nodal network metrics with the insomnia scores in the IS group. Pearson’s correlation analysis was performed with age, gender, educational level, adjusted HAMA score and adjusted HAMD score as unconcerned confounding factors, the AUCs of each network property as an independent variables and the insomnia scores in the IS group as dependent variables. An exploratory threshold matching value of one divided by the number of nodes (1/90 = 0.011) was adopted as a significant threshold for false-positive correction for all analyses.

## Results

### Demographic Data and Clinical Variables

Demographic information and clinical variables from both the IS and NIS groups are provided in Table 1. We found no significant differences between the two groups regarding age (*t*_(91)_ = 0.201, *p* = 0.841), gender (${\text{χ}}_{\text{(1)}}^{\text{2}}$ = 0.532, *p* = 0.466), and educational level (*t*_(91)_ = −1.889, *p* = 0.065). However, both the original HAMD scores and the adjusted HAMD scores exhibited significant differences between the IS and NIS groups (*p* < 0.001, Table 1). The original HAMA scores and the adjusted HAMA scores also differed significantly between groups (*p* < 0.001, Table 1). In addition, the original and adjusted HAMD and HAMA scores in IS group are higher than that in NIS group.

### Group Differences in Global Network Properties

**Figure 1:**
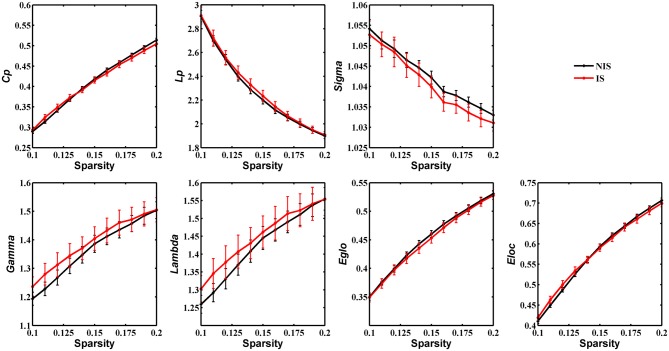
Group comparison of global network topological properties (Cp, Lp, Sigma, Gamma, Lambda, E_glo_ and E_loc_) between the IS and NIS groups (5000 permutations, *p* < 0.05, uncorrected). The small-worldness suggests a small-world topology for brain networks of both the IS and NIS groups. The error bar represents the standard deviation (SD). Cp, clustering coefficient; Lp, characteristic path length; E_glo_, global efficiency; E_loc_, local efficiency; IS, healthy participants with insomnia symptoms; NIS, healthy participants without insomnia symptoms.

Statistical analysis was performed to detect distinctions in the global organization of brain structural networks between the IS and NIS groups. Both groups showed prominent small-world properties for all threshold values from 0.1 to 0.2 (Figure 1), suggesting that the small-world architecture of the human brain is robust to brain aberrations or disorders (Achard et al., 2006). Importantly, the IS group exhibited an increased local efficiency and a decreased global efficiency in the anatomic brain networks as compared with NIS group (Supplementary Table S2), indicating an insomnia-related shift in the topology toward regular networks. However, no statistically significant differences between groups were revealed in regard to the measures of global properties of structural networks (Figure 1 and Supplementary Figure S1, Supplementary Table S2).

### Alterations in Nodal Network Properties in Healthy Participants with Insomnia Symptoms

**Figure 2:**
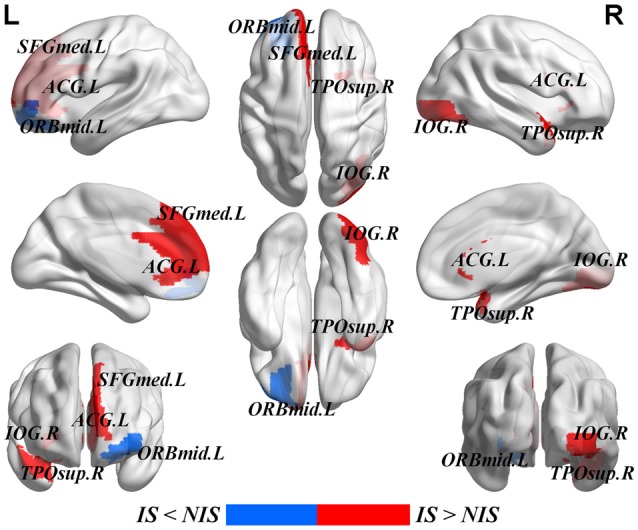
Brain areas with altered nodal betweenness centrality, nodal degree and nodal efficiency in IS group. Group comparisons were based on permutation tests (5000 permutations, *p* < 0.05, uncorrected, controlling for the age, gender, educational level, adjusted HAMA score and adjusted HAMD score). Colored brain areas displayed the significantly aberrant nodal network properties in IS group. The red and blue represent significantly increased and decreased nodal topology in IS group as compared with NIS group, respectively. The more detailed information were showed in Table 2. L, left; R, right; IS, healthy participants with insomnia symptoms; NIS, healthy participants without insomnia symptoms; IOG, inferior occipital gyrus; TPOsup, temporal pole: middle temporal gyrus; ORBmid, middle frontal gyrus, orbital part; SFGmed, superior frontal gyrus, medial; ACG, anterior cingulate gyrus. Figures were visualized based on the BrainNet Viewer software (https://www.nitrc.org/projects/bnv/).

Table 2 provides the results of statistical comparisons of the nodal properties (nodal betweenness centrality, nodal degree and nodal efficiency) between the IS and NIS groups (*p* < 0.05, uncorrected). In comparison with the NIS group, the IS group showed significantly stronger nodal betweenness centrality over two brain regions (the right inferior occipital gyrus (IOG.R) and the right temporal pole: middle temporal gyrus (TPOsup.R)), as well as significantly larger nodal degree in one region (the IOG.R) and significantly higher nodal efficiency over two regions (the left anterior cingulate gyrus (ACG.L) and left superior frontal gyrus, medial (SFGmed.L); Figure 2 and Table 2). In addition, the subjects in the IS group exhibited decreased nodal efficiency in the orbital part of left middle frontal gyrus (ORBmid.L; Figure 2 and Table 2).

**Table 2:** Brain regions showing abnormal nodal network properties in IS as compared with NIS.

Brain regions	Nodal property	IS	NIS	*p*-value
IOG.R	BC	0.05 ± 0.10	0.02 ± 0.05	0.011*
TPOsup.R	BC	0.01 ± 0.02	0.01 ± 0.02	0.042
IOG.R	Deg	84.35 ± 72.31	65.77 ± 58.06	0.029
ORBmid.L	E_nodal_	2.29 ± 2.38	3.67 ± 3.02	0.017
SFGmed.L	E_nodal_	5.97 ± 1.43	5.57 ± 1.81	0.047
ACG.L	E_nodal_	6.69 ± 0.67	6.55 ± 1.34	0.042

Group comparisons: permutation tests (5000 permutations, *p* < 0.05, controlling for the age, gender, educational level, adjusted HAMA score and adjusted HAMD score). Data are reported as mean ± SD for the AUC of the nodal network properties over the range of 0.1 ≤ *S* ≤ 0.2 with an interval of 0.01. IOG, inferior occipital gyrus; TPOsup, temporal pole: middle temporal gyrus; ORBmid, middle frontal gyrus, orbital part; SFGmed, superior frontal gyrus, medial; ACG, anterior cingulate gyrus; IS, healthy participants with insomnia symptoms; NIS, healthy participants without insomnia symptoms. L, left; BC, nodal betweenness centrality; Deg, nodal degree; E_nodal_, nodal efficiency. *Reported results are significant for *p* < 1/90 based on false positive correlation for multiple comparisons.

### Insomnia Was Associated with Nodal Structural Connectivity Topology of Areas Involved in Salience and Default-Mode Networks

**Figure 3:**
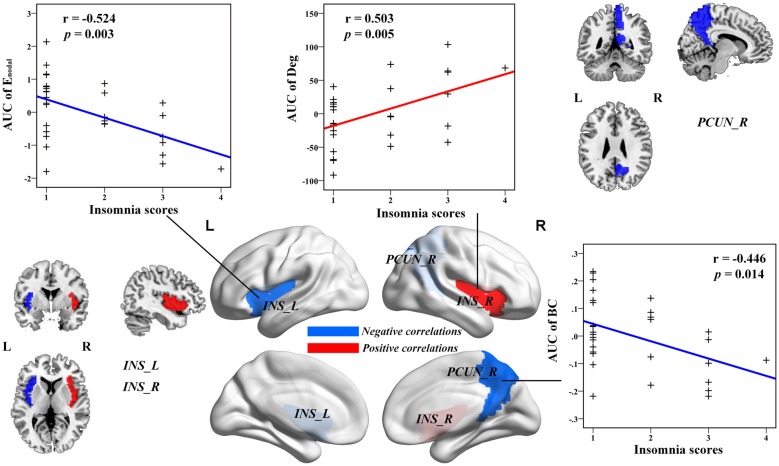
The Pearson correlation between the AUC of the nodal network properties with insomnia scores in healthy participants with insomnia symptoms (*p* < 0.05, uncorrected, controlling for the age, gender, educational level, adjusted HAMA scores and adjusted HAMD score). The AUC of each nodal topology was computed over the range of 0.1 ≤ *S* ≤ 0.2 with an interval of 0.01. The red color stands for the positive correlation while the blue color reveals the negative correlation. For more detailed information see Table 3. L, left; R, right; AUC, area under the curve; BC, nodal betweenness centrality; E_nodal_, nodal efficiency; INS, insula; PCUN, precuneus.

Multiple linear regression analyses revealed no significant correlations between the global network topology and insomnia scores in the IS group. However, the nodal efficiency in the left insula (INS.L) of the salience network showed a negative correlation with insomnia scores (*p* < 0.011, false positive correction; Figure 3 and Table 3). In addition, the nodal betweenness centrality and the nodal degree of the right postcentral gyrus (PoCG.R) exhibited significant negative correlations with insomnia scores (*p* < 0.011, false positive correction; Table 3). Furthermore, the nodal betweenness of the right precuneus (PCUN.R) in the default-mode network also showed significant negative correlations with insomnia scores (*p* < 0.05, uncorrected; Figure 3 and Table 3). Importantly, the nodal betweenness centrality of the left heschl gyrus (HES.L; Table 3) and the nodal degree of the right insula (INS.R; Figure 3 and Table 3) showed significant positive relationships with insomnia scores (*p* < 0.011, false positive correction). In particular, the nodal network properties from several brain regions were also significantly related with insomnia scores, including the right middle temporal gyrus (MTG.R), IOG.R, left superior parietal gyrus (SPG.L), and right HES (HES.R; *p* < 0.05, uncorrected; Table 3).

**Table 3:** Significant correlations between nodal network properties and the insomnia scores in IS group.

Brain regions	Nodal topology	*r-*value	*p*-value
**Negative correlations between nodal topology with insomnia scores**			
PoCG.R	BC	−0.455	0.011*
PCUN.R	BC	−0.446	0.014
MTG.R	BC	−0.381	0.038
IOG.R	Deg	−0.382	0.038
PoCG.R	Deg	−0.466	0.010*
SPG.R	Deg	−0.376	0.041
INS.L	E_nodal_	−0.524	0.003*
SPG.L	E_nodal_	−0.393	0.032
**Positive correlations between nodal topology with insomnia scores**			
HES.L	BC	0.461	0.010*
INS.R	Deg	0.503	0.005*
HES.R	E_nodal_	0.440	0.015

Pearson correlation analyses were corrected by controlling for age, gender, educational level, adjusted HAMA score and adjusted HAMD score. The AUC of the nodal topology was computed over the range of 0.1 ≤ *S* ≤ 0.2 with an interval of 0.01. R, right; L, left; AUC, area under the curve; BC, nodal betweenness centrality; Deg, nodal degree; E_nodal_, nodal efficiency; PoCG, postcentral gyrus; PCUN, precuneus gyrus; MTG, middle temporal gyrus; IOG, inferior occipital gyrus; INS, insula; SPG, superior parietal gyrus; HES, heschl gyrus; IS, healthy participants with insomnia symptoms. *Reported results are significant for *p* < 1/90 based on false positive correlation for multiple comparisons.

## Discussion

This study examined the topology organization of WM networks in insomnia using DTI tractography and graph theory analysis. We discovered that: (1) both the two groups exhibited optimized small-world organization with respect to their WM structural networks; (2) the IS group showed a lower global efficiency and a higher local efficiency than that of the NIS group, illustrating an insomnia-related shift of the topology towards regular networks; (3) the IS group manifested altered nodal brain structural network properties (nodal betweenness centrality, nodal degree and nodal efficiency) in fronto-limbic pathways including the SFGmed.L, the ORBmid.L and the ACG.L; and (4) the salience and default-mode networks showed correlations with insomnia scores. The insomnia scores were negatively associated with the nodal efficiency of the INS.L of the salience network and the nodal betweenness centrality of the PCUN.R of the default-mode network. These findings revealed large-scale topological organization substrates of the structural network, which could provide novel tools for better understanding of the neural circuitry that underlies insomnia. Our results were not explained by gender, age or educational level, which were controlled for during the group comparison analysis. The current results also removed the influence of adjusted HAMD scores and adjusted HAMA scores, suggesting that insomnia rather than depression or anxiety is associated with an altered topology of the fronto-limbic and salience network and default-mode network structural connectivity.

The small-worldness is an important topological property that demonstrates two fundamental organizations of functional segregation as well as functional integration. Functional segregation is associated with specialized processing within densely interconnected brain regions, and functional integration characterizes the ability of information communication between distributed brain areas (Rubinov and Sporns, 2010; Bullmore and Bassett, 2011). Interestingly, we demonstrated that both the healthy participants with IS and those without IS showed economic small-world topology with respect to their large-scale brain WM structural connectivity networks.

However, despite the common small-world topology organizations, the IS and NIS groups exhibited significant differences in nodal topological characteristics. Specifically, increased nodal properties in the IS group were identified in mainly the frontal and temporal lobes (e.g., the SFGmed.L and the TPOsup.R) compared with the NIS group (Table 2). In addition, the nodal network properties in MTG.R in the IS group also showed significant correlations with insomnia scores. The frontal regions are known to play important roles in memory, executive functions and emotion processing (Stuss and Alexander, 2000; Baddeley, 2003). A whole-brain voxel-based morphometry (VBM) study on 24 insomnia patients revealed reduced volume in the left orbitofrontal cortex (Altena et al., 2010). Drummond et al. (2013) demonstrated that individuals with insomnia exhibited decreased activations in the frontal regions when performing working memory tasks. In addition, Li C. et al. (2016) suggested that the insomnia patients had decreased amplitude of low-frequency fluctuation (ALFF) values in the left orbitofrontal cortex and right middle frontal gyrus during resting-state.

Another ALFF study showed significantly decreased amplitudes in the prefrontal cortex and default-mode network sub-regions (Zhou et al., 2016). The temporal lobe structures were recognized to be responsible for disturbed sleep or dyssomnia (Van Sweden, 1996). A recent study highlighted that individuals with poor sleep quality showed increased rates of atrophy within the frontal, temporal and parietal regions (Sexton et al., 2014). Another study showed decreased local coherence in the right temporal, parietal and frontal lobe regions in obstructive sleep apnea (OSA) patients (Santarnecchi et al., 2013). More importantly, a DTI study observed widespread WM integrity alteration, which includes axons linking brain structures within the limbic system, frontal and temporal cortices in OSA (Macey et al., 2008). Furthermore, studies based on VBM approaches demonstrated that the gray matter concentration was significantly decreased in both the cortical and subcortical brain regions, including the fronto-parietal cortices, temporal lobe and anterior cingulate cortex in OSA patients compared to healthy volunteers (Joo et al., 2010; Torelli et al., 2011). However, these discrepancies in frontal and temporal lobe analyses could be caused by the different subjects and imaging methods used (e.g., fMRI, EEG, structural MRI and DTI).

The present method was the first to use the DTI tractography to inspect the small-world changes in WM network in insomnia. Our findings indicated that the alterations of WM structural connectivity in frontal and temporal regions could influence information communication and functional integration for insomnia. In addition, increased nodal properties in the IS group were found in the default-mode network region, including the ACG.L. The ACG is the important part of the limbic system, which is implicated in regulating cognitive and emotional processing (Bush et al., 2000). The ventral ACG is also part of the default-mode network (Margulies et al., 2007). The anterior cingulate areas have extensive connections with the INS, prefrontal cortex, amygdala, hypothalamus and brainstem (Margulies et al., 2007; Cersosimo and Benarroch, 2013). Recently, there has been increasing evidence obtained using positron emission tomography and glucose metabolism from fludeoxyglucose shows that the ACG plays an essential role in the regulation of normal sleep, including between sleep and wake, during sleep deprivation, as well as across sleep stages in humans (Braun et al., 1997; Nofzinger et al., 1997, 2002; Thomas et al., 2000). In addition, using VBM, Winkelman et al. (2013) have found increased rostral ACG in PI patients compared to good-sleeper controls. Using graph theoretical analysis, our findings indicated WM alterations of the structural connections in the ACG. The increased nodal efficiency in the ACG in insomnia may reflect a compensatory response to repetitive sleep disturbance.

The INS is thought to be a key hub of the salience network (Kelly et al., 2012; Tahmasian et al., 2016) and is responsible for the detection of salience, making decision, emotion judgment, attention modulation, motor/sensory processes and cognition regulation (Menon and Uddin, 2010; Cauda et al., 2012; Uddin, 2015). It can be divided into two core parts: one is the anterior insula which is linked with the frontal and parietal cortex, ACG, and limbic regions. It is mainly responsible for salience detection and other emotional processes. Another is the posterior insula which is associated with the sensorimotor, temporal, premotor and posterior cingulate regions. It plays a critical role in perception processing, emotion regulation, interoception and sensorimotor integration (Cauda et al., 2011, 2012). Previous studies have observed insula abnormalities in insomnia patients. For example, Liu C.-H. et al. (2016) observed a decreased fractional ALFF in the INS, indicating the misperception and hyperarousal during sleep state in insomnia patients. Further, Huang et al. (2012) reported decreased functional connections mainly between the INS and the amygdala as well as between the thalamus and striatum in PI.

Our study also showed that decreased nodal efficiency of the left INS was associated with increased insomnia scores. However, Chen et al. (2014) showed an increased activation in the anterior INS with the salience network in female insomnia patients. Recently, Li et al. (2014) found that the PI group exhibited strong connectivity between the right INS and the bilateral superior parietal lobe. In the current study, we discovered that the increased right INS in the nodal degree was related with increased insomnia scores. These discrepant results may due to a result of different sample size of the insomnia patients, gender distinction, potential confounding variables that were controlled for in different studies, or methodological differences. Our findings regarding different changes in the left INS and right INS may provide new evidence that they play different roles in information processing in insomnia. We demonstrated that the INS could be an important neural marker for the hyperarousal pathophysiology underlying insomnia. Taken together, our findings suggest that IS may disrupt the role of the INS in maintaining the functions of alertness and cognitive processing.

Some limitations need to be considered. First, we did not use the Pittsburgh Sleep Quality Index or Duke structured interview to measure the IS. We applied the three-item sleep subscale based on HAMD-17 instead since it is better associated with sleep diaries (Manber et al., 2005). Second, the false-positive correction (1/number of regions) was used in this study, which was not as conservative as the false discovery rate (FDR) correction. Third, we divided the whole brain into 90 sub-regions based on the AAL atlas to construct the brain large-scale structural network. However, previous studies suggest that different parcellation strategies may result in distinct network topological properties (Fornito et al., 2010; Sanabria-Diaz et al., 2010). Therefore, it is necessary to apply a more precise parcellation strategy to provide the information for the brain network topology alterations in insomnia. Fourth, the voxel size of DTI data was not isotropic in the present study which may cause underestimate FA values in brain regions with crossing fibers (Oouchi et al., 2007), which may influence the structural connectivity network. Finally, future studies should employ a high b-value diffusion-weighted acquisition sequence and streamline tractography to estimate structural connectivity and model WM architecture.

## Conclusion

We applied DTI tractography combined with graph theory approaches to explore the abnormalities of topological organization in WM structural networks of subjects with IS. Both the healthy subjects with IS and those without IS showed small-world organization. However, the insomnia group showed altered regional network properties in the fronto-limbic system. The salience and default-mode networks were also strongly linked with insomnia. Our results demonstrated a disrupted WM network integrity and thus provided structural insights into the insomnia connectome. Importantly, certain structural networks can provide important implications for understanding the brain structural connectome in insomnia.

## Author Contributions

F-ML, JD, C-HL, H-FC, M-XH and ZY conceived and designed the experiments. C-HL, S-LL, L-RT and C-LT acquired the data, which F-ML, HC and ZY analyzed. F-ML, JD, TAC, Y-TX and ZY wrote the article, which all authors reviewed and approved for submission.

## Conflict of Interest Statement

The authors declare that the research was conducted in the absence of any commercial or financial relationships that could be construed as a potential conflict of interest.

## Footnotes

^1^www.fmrib.ox.ac.uk/fsl
