# Supplementary material for: Diffusion Tensor Imaging Tractography Reveals Disrupted White Matter Structural Connectivity Network in Healthy Adults with Insomnia Symptoms
Source: Front Hum Neurosci. 2017 Nov 30;11:583. doi: 10.3389/fnhum.2017.00583 (PMC5715269; doi:10.3389/fnhum.2017.00583)
Supplement: Supplementary file 1 [file Presentation_1.pdf]

Supplementary Material

Diffusion Tensor Imaging Tractography Reveals Disrupted White Matter Structural Connectivity Network in Healthy Adults with Insomnia Symptoms

Feng-Mei Lu [a,#], Jing Dai [b,#], Tania Alexandra Couto [a], Chun-Hong Liu [c, d], Heng Chen [e], Shun-Li Lu [d], Li-Rong Tang [d], Chang-Le Tie [d], Hua-Fu Chen [e], Man-Xi He [b], Yu-Tao Xiang [a], Zhen Yuan [a,*]

Network Analysis

Small-world network parameters

The functional connectivity network can be evaluated using graph theory analysis in which a network comprised the nodes and edges. For an N × N (N = 90 indicates 90 nodes in the present study) binary undirected graph *G*, the topological properties were defined on the basis of the following graph construction:

$$e_{ij} = \begin{cases} 1, & if\ |z_{ij}| \geq T \\ 0, & otherwise \end{cases},$$

If the absolute z_{ij} (the Fisher *r*-to-*z* of the partial correlation coefficient between node i and node j) exceeds a given threshold *T*, an undirected edge is said to exist; otherwise it does not exist.

The clustering coefficient of a node i is defined as the ratio of the number of existing connections among the node's neighbors to the number of all possible connections in the subgraph G_i (Onnela et al., 2005) and is expressed as:

$$C_i = \frac{E_i}{K_i\,(K_i - 1)/2},$$

in which E_i and K_i denote the number of edges and nodes respectively in the

subgraph G_i. Then the clustering coefficient of a functional connectivity network is the average of the clustering coefficients of all nodes:

$$C_p = \frac{1}{N}\sum_{i\in G} C_i,$$

it measures the local interconnectivity of a network.

The mean shortest path length of a node i is defined as:

$$L_i = \frac{1}{N-1}\sum_{i\neq j\in G} min|L_{ij}|,$$

where $min|L_{ij}|$ is the absolute shortest path length (i.e. the smallest number of edges traversed between two nodes) between node i and node j. The mean shortest path length of a network is then the average of the shortest path lengths between the nodes:

$$L_p = \frac{1}{N}\sum_{i\in G} L_i,$$

The normalized clustering coefficient $\gamma = \frac{C_p}{C_{random}}$ and normalized characteristic path length $\lambda = \frac{L_p}{L_{random}}$ were computed, where C_p and L_p indicate the mean clustering coefficient and shortest path length of the functional connectivity network, respectively. C_{random} and L_{random} represent the mean clustering coefficient and shortest path length of 100 matched random networks that preserved the same number of nodes, edges, and degree distribution as the real network (Sporns and Zwi, 2004; Ding et al., 2011). Typically, a small-word network meet the conditions of $\gamma > 1$ and $\lambda \approx 1$, and therefore, the small-wordness scalar $\sigma = \lambda/\gamma$ will be more than 1.

Efficiency of small-world networks

Network efficiency can be measured by global efficiency, E_{glo}, local efficiency, E_{loc} and nodal efficiency, E_{nodal}, E_{glo} and E_{loc} described the ability of information transmission of a network at the global and local level, respectively. The global

efficiency E_{glo} of a network is the inverse of the harmonic mean of the shortest path length between each pair of nodes (Latora and Marchiori, 2001; Achard and Bullmore, 2007):

$$E_{glo} = \frac{1}{N(N-1)} \sum_{i \neq j \in G} \frac{1}{min|L_{ij}|},$$

where $min|L_{ij}|$ is the absolute shortest path length between node i and node j in network G. It indicates the capability of parallel information transfer through the whole network.

The nodal efficiency E_{nodal} of a node i is calculated as:

$$E_{nodal}(\mathrm{i}) = \frac{1}{N-1} \sum_{j,k \in G} \frac{1}{min|L_{jk}|},$$

The local efficiency E_{loc} denoted the mean of all the local efficiencies of the nodes in subgraph G_i which is defined as:

$$E_{loc} = \frac{1}{N} \sum_{i \in G} E_{nodal}(i),$$

where $E_{nodal}(i) = E_{glo}(G_i)$. Since the node i is not an element of the subgraph G_i, the local efficiency can also be considered as a measure of the fault tolerance of the network, suggesting how well each subgraph exchanges information when the node i was eliminated (Achard and Bullmore, 2007).

The integrated area under curve (AUC) of a network metric Y was computed over the sparsity threshold range from S_1 to S_n with interval of ΔS, which was expressed as:

$$Y^{AUC} = \sum_{k=1}^{n-1} [Y(S_k) + Y(S_{k+1})] \times \Delta S/2.$$

Tables and Figures

Table S1. Cortical and subcortical regions of interest defined in this study.

Region Name	Abbreviation	Region Name	Abbreviation
Precentral gyrus	PreCG	Lingual gyrus	LING
Superior frontal gyrus, dorsolateral	SFGdor	Superior occipital gyrus	SOG
Superior frontal gyrus, orbital	ORBsup	Middle occipital gyrus	MOG
Middle frontal gyrus	MFG	Inferior occipital gyrus	IOG
Middle frontal gyrus, orbital	ORBmid	Fusiform gyrus	FFG
Inferior frontal gyrus, opercular part	IFGoperc	Postcentral gyrus	PoCG
Inferior frontal gyrus, triangular	IFGtriang	Superior parietal gyrus	SPG
Inferior frontal gyrus, orbital	ORBinf	Inferior parietal gyrus	IPG
Rolandic operculum	ROL	Supramarginal gyrus	SMG
Supplementary motor area	SMA	Angular gyrus	ANG
Olfactory cortex	OLF	Precuneus	PCUN
Superior frontal gyrus, medial	SFGmed	Paracentral lobule	PCL
Superior frontal gyrus, medial orbital	ORBsupmed	Caudate nucleus	CAU
Gyrus rectus	REC	Putamen	PUT
Insula	INS	Pallidum	PAL
Anterior cingulate gyri	ACG	Thalamus	THA
Median cingulate gyri	DCG	Heschl gyrus	HES

Posterior cingulate gyrus	PCG	Superior temporal gyrus	STG
Hippocampus	HIP	Superior temporal gyrus: temporal pole	TPOsup
Parahippocampal gyrus	PHG	Middle temporal gyrus	MTG
Amygdala	AMYG	Middle temporal gyrus: temporal pole	TPOmid
Calcarine fissure	CAL	Inferior temporal gyrus	ITG
Cuneus	CUN		

The regions are listed according to a prior AAL atlas (Tzourio-Mazoyer et al., 2002).

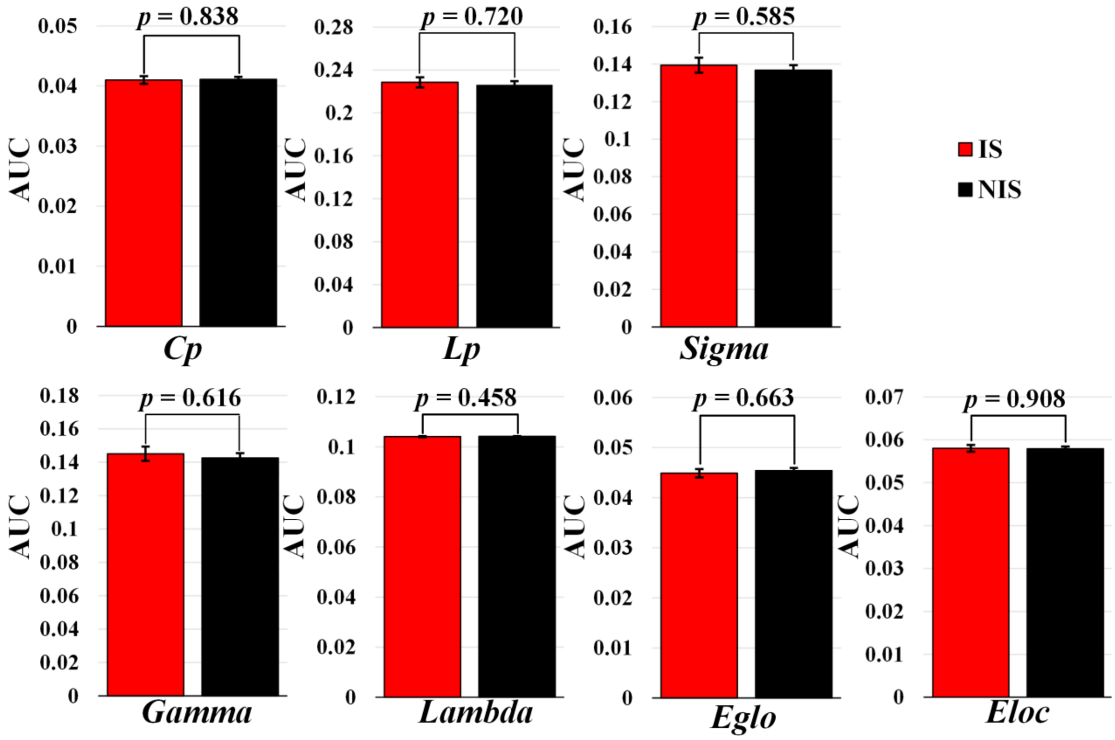

Figure. S1 Group comparison of the AUC of global network topological properties (Cp, Lp, Sigma, Gamma, Lambda, E_{glo}, and E_{loc}) between the IS and NIS groups. There was no significant differences between two groups (5,000 permutations, $p < 0.05$, uncorrected). The error bar represents the standard error (SE). Cp, clustering

coefficient; Lp, characteristic path length; E_{glo}, global efficiency; E_{loc}, local efficiency; IS, healthy participants with insomnia symptoms; NIS, healthy participants without insomnia symptoms.

Table S2. Global network properties in IS as compared with NIS.

**Global topology**	**IS**	**NIS**	***p*-value**
Small-worldness (Sigma)	0.139 ± 0.0040	0.136 ± 0.0027	0.578
Normalized characteristic path length (Gamma)	0.229 ± 0.0043	0.226 ± 0.0029	0.618
Normalized clustering coefficient (Lambda)	0.104 ± 0.0002	0.104 ± 0.0001	0.454
Characteristic clustering coefficient (Cp)	0.041 ± 0.0006	0.041 ± 0.0004	0.847
Characteristic path length (Lp)	0.229 ± 0.0047	0.226 ± 0.0033	0.710
Global efficiency (E_{glo})	0.044 ± 0.0008	0.045 ± 0.0006	0.670
Local efficiency (E_{loc})	0.058 ± 0.0008	0.057 ± 0.0005	0.912

Group comparison of global network topological properties between the IS and NIS groups (5,000 permutations, $p < 0.05$, uncorrected). Data are reported as mean ± SD for the AUC of the network properties over the range of $0.1 \leq S \leq 0.2$ with an interval of 0.01. IS, healthy participants with insomnia symptoms; NIS, healthy participants without insomnia symptoms.

Reference

Achard, S., and Bullmore, E. (2007). Efficiency and cost of economical brain functional networks. *PLoS Computational Biolology* 3(2)**,** e17. doi: 10.1371/journal.pcbi.0030017.

Ding, J.R., Liao, W., Zhang, Z., Mantini, D., Xu, Q., Wu, G.R., et al. (2011). Topological fractionation of resting-state networks. *PLoS ONE* 6(10)**,** e26596. doi: 10.1371/journal.pone.0026596.

Latora, V., and Marchiori, M. (2001). Efficient behavior of small-world networks. *Physical Review Letters* 87(19)**,** 198701.

Onnela, J.P., Saramaki, J., Kertesz, J., and Kaski, K. (2005). Intensity and coherence of motifs in weighted complex networks. *Physical Review E Statistical Nonlinear Soft Matter Physics* 71(6 Pt 2)**,** 065103.

Sporns, O., and Zwi, J.D. (2004). The small world of the cerebral cortex. *Neuroinformatics* 2(2)**,** 145-162. doi: 10.1385/NI:2:2:145.

Tzourio-Mazoyer, N., Landeau, B., Papathanassiou, D., Crivello, F., Etard, O., Delcroix, N., et al. (2002). Automated anatomical labeling of activations in SPM using a macroscopic anatomical parcellation of the MNI MRI single-subject brain. *Neuroimage* 15(1)**,** 273-289. doi: 10.1006/nimg.2001.0978.
